# Supplementary material for: Comparative genomics provides new insights into the diversity, physiology, and sexuality of the only industrially exploited tremellomycete: Phaffia rhodozyma
Source: BMC Genomics. 2016 Nov 9;17:901. doi: 10.1186/s12864-016-3244-7 (PMC5103461; doi:10.1186/s12864-016-3244-7)
Supplement: Additional file 6: — List of orphan genes with links to PFAM (related to Additional file 1: Table S1). (ZIP 1428 kb) [file 12864_2016_3244_MOESM6_ESM.zip › PFAM_UNIQUE/G03007_P.html]

#                                                                            --- full sequence --- -------------- this domain -------------   hmm coord   ali coord   env coord
  
# target name        accession   tlen query name           accession   qlen   E-value  score  bias   #  of  c-Evalue  i-Evalue  score  bias  from    to  from    to  from    to  acc description of target
  
#------------------- ---------- ----- -------------------- ---------- ----- --------- ------ ----- --- --- --------- --------- ------ ----- ----- ----- ----- ----- ----- ----- ---- ---------------------
  
G03007\_P             -            364 CDC27                PF09507.5    430       5.5    4.8   6.8   1   1     0.048       7.5    4.3   6.8   129   201   122   193   107   217 0.35 - DNA polymerase subunit Cdc27  
G03007\_P             -            364 DDHD                 PF02862.12   227       2.2    6.7   6.9   1   1    0.0081       3.2    6.1   6.9    80   168    85   191    76   206 0.43 - DDHD domain  
G03007\_P             -            364 DUF1675              PF07897.6    284       7.7    4.9  11.9   1   1     0.015        12    4.4  11.9   139   219   123   202   108   215 0.32 - Protein of unknown function (DUF1675)  
G03007\_P             -            364 DUF2413              PF10310.4    444      0.55    7.6   3.8   1   1     0.002      0.76    7.2   3.8    88   180   119   208   112   256 0.70 - Protein of unknown function (DUF2413)  
G03007\_P             -            364 DUF4614              PF15391.1    182      0.88    8.0  12.9   1   1    0.0037         2    6.8  12.9    22    88   122   186   111   214 0.52 - Domain of unknown function (DUF4614)  
G03007\_P             -            364 DUF566               PF04484.7    313      0.25    9.5  10.6   1   1    0.0013      0.34    9.0  10.6    46   123   123   202    85   216 0.69 - Family of unknown function (DUF566)  
G03007\_P             -            364 DUF966               PF06136.8    337       1.1    7.3  11.1   1   1    0.0041       1.5    6.9  11.1   102   184   106   190    78   208 0.61 - Domain of unknown function (DUF966)  
G03007\_P             -            364 Fasciclin            PF02469.17   128   2.5e-05   23.0   1.7   1   2   7.5e-05      0.11   11.1   1.5    16    76    21   107    11   181 0.64 - Fasciclin domain  
G03007\_P             -            364 Fasciclin            PF02469.17   128   2.5e-05   23.0   1.7   2   2   0.00039      0.58    8.8   0.0    24   125   277   360   250   363 0.79 - Fasciclin domain  
G03007\_P             -            364 FNIP\_N               PF14636.1    131       1.5    8.0   6.0   1   1   0.00091       2.7    7.1   6.0    36    83   133   193   108   218 0.48 - Folliculin-interacting protein N-terminus  
G03007\_P             -            364 Herpes\_BLLF1         PF05109.8    830       9.2    2.7  20.7   1   1     0.023        11    2.5  20.7   443   556    29   187     3   288 0.57 - Herpes virus major outer envelope glycoprotein (BLLF1)  
G03007\_P             -            364 Herpes\_UL32          PF06070.6    839      0.37    7.9   7.4   1   1   0.00052      0.44    7.6   7.4   646   731   112   193    54   224 0.75 - Herpesvirus large structural phosphoprotein UL32  
G03007\_P             -            364 LAP1C                PF05609.7    465       5.3    4.2   8.6   1   1    0.0041       8.3    3.6   8.6   120   195   124   199   109   215 0.74 - Lamina-associated polypeptide 1C (LAP1C)  
G03007\_P             -            364 Macoilin             PF09726.4    697     0.071   10.0  11.5   1   1   0.00063     0.083    9.8  11.5   287   385    93   198    11   229 0.58 - Transmembrane protein  
G03007\_P             -            364 Med3                 PF11593.3    379     0.034   12.0  16.9   1   1   0.00014     0.049   11.5  16.9   123   209   118   205   108   246 0.67 - Mediator complex subunit 3 fungal  
G03007\_P             -            364 MGC-24               PF05283.6    186       6.4    5.2  15.2   1   1     0.014        16    3.8  15.2    88   152   126   188   107   220 0.54 - Multi-glycosylated core protein 24 (MGC-24)  
G03007\_P             -            364 PAP1                 PF08601.5    347      0.44    8.8  11.8   1   1    0.0016      0.53    8.6  11.8    95   176   118   191    31   234 0.51 - Transcription factor PAP1  
G03007\_P             -            364 Peptidase\_S64        PF08192.6    695       1.7    5.5   7.6   1   1    0.0068       2.7    4.9   7.6    29   118   121   207   106   248 0.56 - Peptidase family S64  
G03007\_P             -            364 Period\_C             PF12114.3    195     0.038   12.3  13.6   1   1    0.0001     0.067   11.4  13.6     7    78   123   193   114   259 0.64 - Period protein 2/3C-terminal region  
G03007\_P             -            364 Pol\_alpha\_B\_N        PF08418.5    253         5    5.3  10.3   1   1     0.023       7.5    4.7  10.3    97   175   119   187    70   210 0.47 - DNA polymerase alpha subunit B N-terminal  
G03007\_P             -            364 SerH                 PF06873.6    403       6.6    3.9  10.3   1   1    0.0044       8.8    3.5  10.3   179   245   123   188    46   213 0.62 - Cell surface immobilisation antigen SerH  
G03007\_P             -            364 SOBP                 PF15279.1    306    0.0068   15.5   0.4   1   1   6.2e-05    0.0085   15.2   0.4    81   163   118   205   102   287 0.64 - Sine oculis-binding protein  
G03007\_P             -            364 SOG2                 PF10428.4    445       4.6    4.4  14.2   1   1     0.028         6    4.1  14.2   201   290    79   182    36   206 0.57 - RAM signalling pathway protein  
G03007\_P             -            364 SprA-related         PF12118.3    295      0.58    8.1   7.3   1   1    0.0051      0.71    7.8   7.3     8    70   125   189    47   208 0.49 - SprA-related family  
G03007\_P             -            364 TCP                  PF03634.8    138       3.7    6.6   6.3   1   1     0.003         6    5.9   6.3    60   130   121   191   103   205 0.39 - TCP family transcription factor
